# Genome-Wide Identification of Melon Single-Nucleotide Polymorphisms and Structural Variations Associated with Resistance to *Fusarium oxysporum* f. sp. *melonis* Race 1.2

**DOI:** 10.3390/plants15142205

**Published:** 2026-07-19

**Authors:** Abolfazl Bozorgmehr, Mohammad Sadegh Sabet, Mohammad Ali Malboobi, Stefano Pavan, Chiara Delvento, Ahmad Moieni

**Affiliations:** 1 Department of Plant Genetics and Breeding, College of Agriculture, Tarbiat Modares University, Tehran P.O. Box 14115-336, Iran; 2 Department of Plant Biotech, National Institute of Genetic Engineering and Biotechnology, Tehran P.O. Box 14965-161, Iran; 3 Department of Soil, Plant and Food Science, University of Bari Aldo Moro, Via Amendola 165/A, 70126 Bari, Italy

**Keywords:** plant pathogen interaction, population structure, quantitative trait, genetic diversity, *Cucumis melo*

## Abstract

Fusarium wilt, caused by *Fusarium oxysporum* f. sp. *melonis* (FOM), is a main disease of melon (*Cucumis melo* L.). FOM 1.2 is the most widespread and detrimental variant of FOM, causing substantial economic losses under severe disease conditions. Current information suggests that resistance to race 1.2 (FOM 1.2) is controlled by multiple recessive genes and is strongly influenced by the environment. Therefore, identifying genetic polymorphisms within diverse melon populations is essential to elucidate the loci and putative candidate genes associated with resistance. The objective of this investigation was to identify single-nucleotide polymorphism (SNP) and structural variant (SV) markers associated with FOM 1.2 resistance utilizing a panel of 160 genotypes through a genome-wide association study (GWAS). Phenotypic evaluation was performed two weeks after sowing, at the first-true-leaf stage, on 2400 individual plants inoculated by the root dip method with a concentration of about 10^6^ spores/mL. Biochemical and disease-related traits, including area under disease progress curve (AUDPC), disease severity index (DSI), standardized AUDPC (SAUDPC), latent period (LP), catalase, peroxidase activity, and ascorbate peroxidase activity were measured 35 days after inoculation. PCA identified eighty-three individual melon plants with a broad range of disease-response variation. Genotyping-by-sequencing (GBS) was conducted on these plants, resulting in the identification of 737,435 SNPs and 75,133 SVs. Evaluation of the population structure outlined four genetic groups, including one associated with germplasm highly resistant to FOM 1.2. We used SNP data to describe linkage disequilibrium (LD), which was estimated to decay at 14 kb, on average. A GWAS was performed using the Bayesian information and linkage-disequilibrium iteratively nested keyway (BLINK) method, which revealed nine SNPs significantly associated with several disease indices, namely ascorbate peroxidase activity, AUDPC, catalase, peroxidase activity, rAUDPC, and SAUDPC. Also, eight SVs were associated with AUDPC and relative area under disease progress curve (rAUDPC), including translocation and deletion types. In addition, GWAS using the fixed and random model circulating probability unification (FarmCPU) method unveiled thirteen SVs associated with rAUDPC, peroxidase activity and ascorbate peroxidase activity, including translocation and inversion types. According to the performed models of GWAS, several significant SNPs and SVs, associated with putative candidate genes, including multidrug resistance-associated protein 6 (MRP6), LOB domain-containing protein 15 (LBD15), phosphomannomutase, and NADH-ubiquinone oxidoreductase B8 subunit, which may be involved in FOM 1.2 resistance. However, these findings represent a preliminary genome-wide survey and require further validation using high-coverage or long-read sequencing approaches. The results provide remarkable insights into the genetic control of FOM 1.2 resistance and valuable information for the implementation of the putative molecular markers identified in this study in melon breeding programs.

## 1. Introduction

Melon (*Cucumis melo* L. Cucurbitaceae) is a diploid (2*n* = 2*x* = 24) plant species with an estimated genome size of 375 Mbp and one of the most important horticultural crops worldwide [1] with an average production exceeding 28.6 million tons per year [2]. *Fusarium oxysporum* f. sp. *melonis* (FOM) (Leach & Currence) Snyder & Hansen [3] is the causal agent of Fusarium wilt, one of the most destructive diseases of melon, causing nearly complete yield loss under severe infection conditions. Current control methods are largely ineffective; as chemical and biological control strategies have shown limited success against FOM in melon. The limited efficacy of chemical treatments is mainly due to the soil-borne nature of the pathogen, the long-term survival of its chlamydospores, and the poor translocation of fungicides into the vascular tissues where the pathogen colonizes. One of the most effective and economical ways to control Fusarium wilt disease is breeding for resistance traits. The use of resistant cultivars represents one of the most cost-effective and sustainable approaches to control FOM. The pathogen can persist for a long time in the soil through chlamydospores that colonize the roots of susceptible hosts [4]. Several commercial varieties of watermelon, melon, and cowpea have been developed that are highly resistant to Fusarium wilt [5,6,7]. Fungi use enzymes like cellulases to degrade plant cell walls. Upon detection of these fungal proteins, plants respond by producing enzyme inhibitors and depositing callose and lignin to strengthen the cell wall [8]. Upon cell and tissue damage, pathogen invasion triggers the production of peroxidases, which generate free reactive oxygen species (ROS) involved in plant defense response [9]. Peroxidases also participate in the polymerization of macromolecules, contributing to cell wall strengthening against pathogen invasion [9]. Catalases have been found to contribute to pathogen virulence by participating in ROS removal [10]. The activity of these and other enzymes may play an important role in determining the level of resistance to FOM 1.2 [11]. Such biochemical responses are closely tied to genetic mechanisms regulating plant resistance to pathogen infection.

Classical genetic studies have identified major resistance genes such as FOM-1 and FOM-2, which confer specific resistance against fungal races 0 and 2 [12]. In addition, the recessive gene FOM-4, linked to FOM-1, confers resistance to races 0 and 2 in the Spanish melon cultivar Tortuga [13]. The Fusarium wilt resistance gene FOM-2 has been cloned using a map-based methodology, and its sequence carries a nucleotide-binding leucine-rich repeat (NBS-LRR) domain that is typically found in plant R genes involved in pathogen recognition and defense signaling [14]. Unfortunately, even with the availability of these genetic sources of resistance, Fusarium wilt remains a significant disease, mainly due to the FOM race 1.2 [15]. Studies show that resistance to FOM 1.2 is controlled by multiple recessive genes and strongly influenced by the environment [16]. According to the previous studies, genetic factors controlling resistance to FOM 1.2 include nine QTLs, two complementary recessive genes, and a major recessive QTL linked to a locus controlling fruit netting [17,18]. Therefore, exploring genetic diversity is essential to identify and map new loci associated with resistance to FOM 1.2, which can provide valuable insights for breeding programs.

Identification of DNA single-nucleotide polymorphisms (SNPs) is ideal for describing genetic diversity, as they occur in large numbers across the genome and possess desirable features such as wide genomic distribution, high reproducibility, and codominant inheritance. The discovery of SNPs in diverse melon botanical groups will allow marker-anchoring to the whole genome sequence (WGS) and provide a better understanding of the genetic control of domestication and diversification [19]. In addition, structural variants (SVs) represent various genomic DNA rearrangements (over 50 bp), such as deletions, insertions, inversions, translocations, and duplications. SVs may play a significant role in plant growth and development [20], as well as in tolerance to environmental stresses and, possibly, pathogens [21,22]. Recent studies have revealed that SNPs and SVs can alter gene expression, regulatory elements, and resistance gene architecture, thereby influencing plant-pathogen interactions and disease resistance. Therefore, precise identification of SNPs and SVs is essential for elucidating the genetic basis of plant defense mechanisms and for accelerating the development of resistant cultivars [23]. For instance, some SVs have been associated with disease resistance to *Cladosporium fulvum* in tomato [24] and copy number variation at the *Rhg1* locus in soybean confers resistance against the soybean cyst nematode (*Heterodera glycines*) [25].

The advent of next-generation sequencing (NGS) prompted genotyping-by-sequencing (GBS) as one of the most convenient NGS-based approaches for simultaneous SNP discovery and genotyping, which is widely used for plant species with or without reference genome sequence information [26,27,28,29,30,31]. In addition to SNP discovery, GBS-derived datasets have also been used to explore genomic variation and identify putative structural variants, such as copy number variations (CNVs), in plant populations. However, due to the reduced-representation nature of GBS, the identification of structural variants using this approach may have a lower resolution compared with that of whole-genome sequencing methods [32]. Therefore, as a flexible and low-cost tool, GBS has been used to map QTLs and genes of interest through high-density linkage mapping and genome-wide association study (GWAS) [33,34,35,36]. GWAS, based on linkage disequilibrium (LD), has emerged as a powerful tool in molecular plant breeding, enabling the identification of molecular markers and candidate genes associated with resistance to major plant diseases. LD decay patterns strongly influence GWAS performance by determining the marker density required for effective association mapping and the resolution at which trait-associated loci can be identified. Populations with faster LD decay generally require a higher marker density to capture associations; however, they provide greater mapping resolution, allowing more precise localization of candidate genomic regions [37].

Here, we performed GWAS using phenotypic data and GBS on a melon germplasm collection, in order to gather genomic information at the population level for resistance to FOM 1.2. In addition to SNP identification, we have also conducted preliminary work on finding the candidate SVs that are possibly associated with resistance to this pathogenic fungus.

## 2. Results

### 2.1. Phenotypic Screening

#### 2.1.1. Validation of Resistance and Susceptibility in Control Genotypes

**Figure 1:**
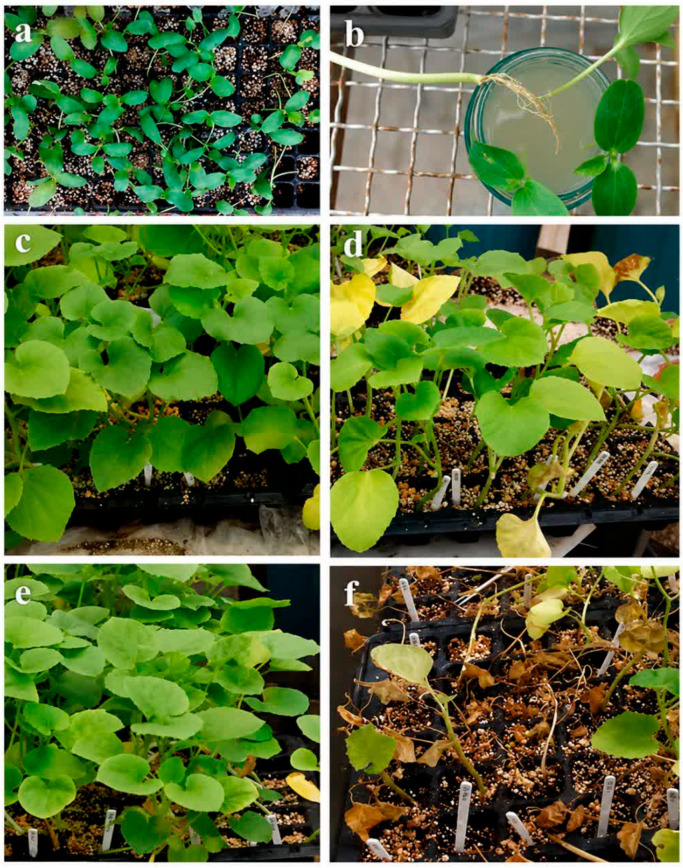
Growth and response of melon plants to Fusarium wilt 35 days after inoculation with the *Fusarium oxysporum* race 1.2 (FOM 1.2). (**a**) At 14 days after planting melon plants (at inoculation stage); (**b**) inoculation of the roots of melon plants with FOM 1.2; (**c**) mock-inoculated (treated with sterile water) cv. Isabelle; (**d**) Isabelle inoculated with FOM 1.2; (**e**) mock-inoculated cv. Charentais-T; (**f**) Charentais-T inoculated with FOM 1.2.

To evaluate the pathogenicity of FOM 1.2, the resistant (Isabelle) and susceptible (Charentais-T) melon genotypes were included as the control plants. Following inoculation, the disease symptoms were observed in both control genotypes. The average disease scores were determined to be 2 in Isabelle as the resistant and 4 in Charentais-T as the susceptible genotypes, respectively (Figure 1a–f).

#### 2.1.2. Phenotypic Evaluation of Melon Genotypes in Response to FOM 1.2

AUDPC, SAUDPC, rAUDPC, DSI, and LP disease-related indices showed significant variation among studied plants in response to FOM 1.2. Among the 2400 evaluated plants, 1.6% were classified as resistant (score 1–2), 24% as susceptible (score 4–5), and 74.4% exhibited intermediate disease severity (score 3) following inoculation with FOM 1.2. These results showed substantial diversity in response to FOM 1.2 disease among the evaluated individual melon plants. According to the results, AUDPC ranged in the studied individuals from 30,660 to 164,140, SAUDPC from 958.12 to 5129.37, rAUDPC from 904.89 to 1136.31, DSI from 4.23 to 19.52, and LP from 9 to 41.

### 2.2. Principal Component Analysis of Melon Response to FOM 1.2

**Figure 2:**
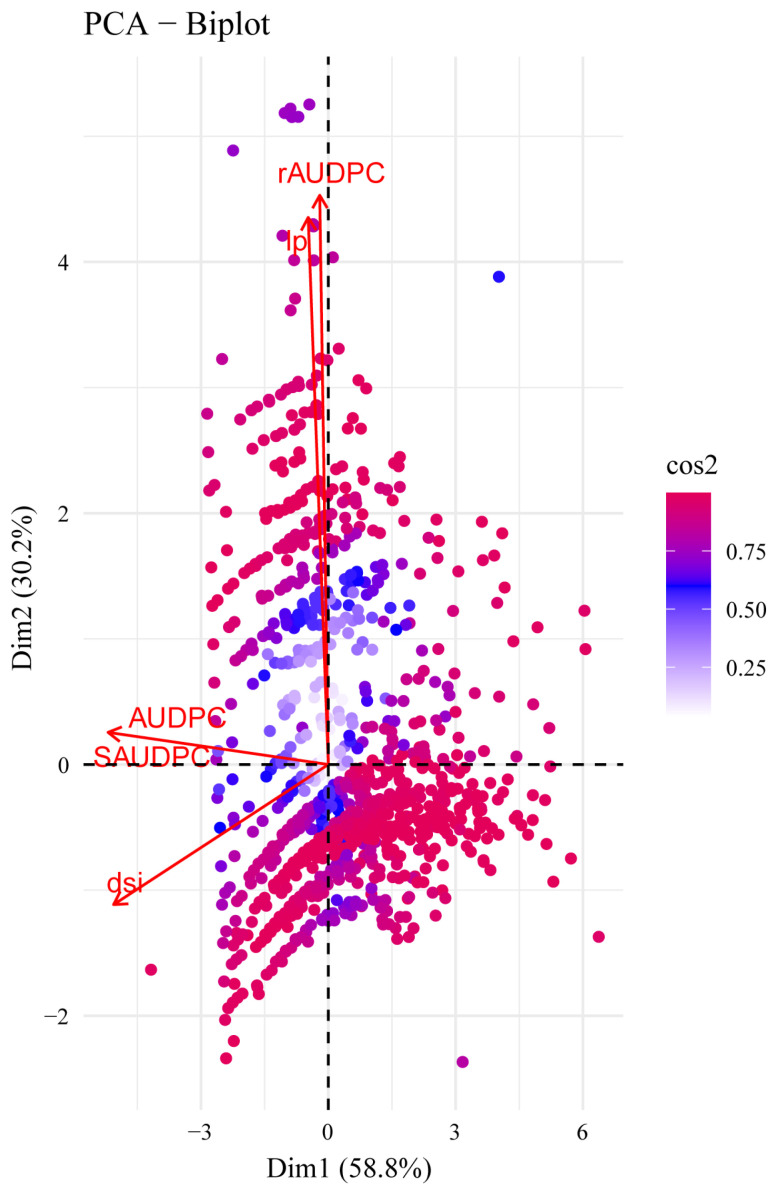
Principal component analysis (PCA) of 2400 individual melon plants inoculated with FOM 1.2 (*Fusarium oxysporum* race 1.2) based on the calculated values for the variables AUDPC, SAUDPC, rAUDPC, DSI, and LP. Dim1 and Dim2 represent the first and the second principal components, explaining the major sources of variation among the genotypes. The color gradient (cos^2^ values) indicates the quality of representation of each variable by the first two PCAs, where higher cos^2^ values (red) indicate better representation of variables on the PCA plot, whereas lower values (blue) indicate weaker representation.

In order to identify the individual plants with a broad range of responses to FOM 1.2, the principal component analysis (PCA) was performed on 2400 melon plants inoculated with FOM 1.2, based on their related values for AUDPC, SAUDPC, rAUDPC, DSI, and LP. The PCA separated the studied plants across a spectrum of responses from susceptible to resistant to FOM 1.2 (Figure 2). The first two principal components (PC1 and PC2) explained 89% of the total phenotypic variation, with PC1 accounting for 58.8% and PC2 for 30.2% (Figure 2). Most of the individual plants with lower PC1 and higher PC2 values displayed resistance to FOM 1.2. The individual plants corresponding to Korean and Japanese genotypes, such as Senari, showed higher resistance to FOM 1.2 in comparison with the others.

The PCA-based evaluation resulted in the identification of eighty-three individual plants from different studied genotypes that encompassed a broad range of responses from resistance to susceptibility. These plants were used for biochemical evaluation, DNA extraction, sequencing, population structure and GWAS analysis. Based on the indices, the individual plants of the Isabelle and Senari2 genotypes were the most resistant to disease FOM 1.2. The lowest AUDPC, SAUDPC, rAUDPC, DSI and the highest LP were observed in an individual plant of the Senari2 genotype with 67.16, 67.16, 19.74, 59.09% AUDPC, SAUDPC, rAUDPC, DSI and 115.78% LP, respectively, as compared to Isabelle (Table S2). In addition, individual plants of the Senari1 and Zard Ghanari Gerd genotypes ranked next in order compared to Isabelle in terms of the mentioned indicators. In contrast, individual plants such as Talebi Resh Baba, Laki Zard, ShahAbadi and Mashhadi exhibited the highest AUDPC, SAUDPC, rAUDPC and DSI together with the lowest LP values, representing the susceptible group. The remaining individuals showed intermediate values across all indices, forming a continuous phenotypic spectrum of response to FOM 1.2 (Table S2).

### 2.3. Physiological Responses of Melon Plants to FOM 1.2 Inoculation

**Figure 3:**
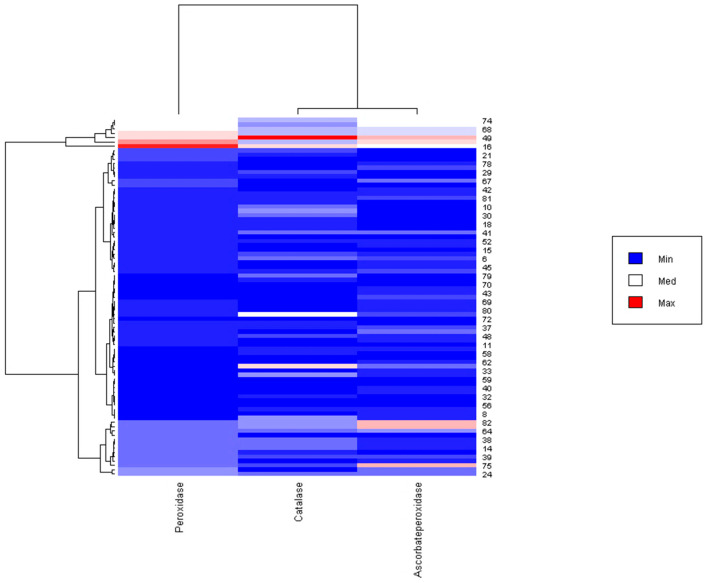
Heatmap of the activities of peroxidase, catalase, and ascorbate peroxidase enzymes in melon plants inoculated with FOM 1.2 (*Fusarium oxysporum* race 1.2). The colors represent normalized enzyme activity levels.

In order to evaluate the physiological response of the studied melon plants to FOM 1.2, the activities of peroxidase, catalase, and ascorbate peroxidase enzymes were evaluated in the eighty-three screened plants through PCA results. The results showed a broad range of disease responses among the studied plans. The dendrogram revealed considerable variation among individual plants, indicating marked variation in their physiological response patterns. The highest peroxidase activity was observed in an individual plant of the Senari1 genotype (48.13 U/mg), whereas the highest activities of catalase and ascorbate peroxidase (0.285 and 1.87 U/mg) were found in an individual plant of the Isabelle genotype (Figure 3 and Table S3). Among the screened individual plants, those belonging to six genotypes, namely Senari1, Kanri kik makuma, TN-92-300, Senari2, TN-62-317, and TN-92-533, were grouped with Isabelle. Individual plants of genotypes such as Gorgab Isoleh, Kharbozeh Zard Moshabak Dorosht, Zarand, INR-C1 and Dastanbo exhibited intermediate peroxidase, catalase, and ascorbate peroxidase activities, whereas the lowest peroxidase activity was detected in an individual plant of genotype Talebi Fyrozan Esfahan (0.685 U/mg). The catalase activity was the lowest in the individual plants of genotypes Khatooni and Kharbozeh Shemam (0.001 U/mg), and the ascorbate peroxidase activity was in an individual plant of Galia Willimorin genotype (0.024 U/mg) (Figure 3 and Table S3).

### 2.4. Correlation of Disease Parameters and Resistance to FOM 1.2

**Figure 4:**
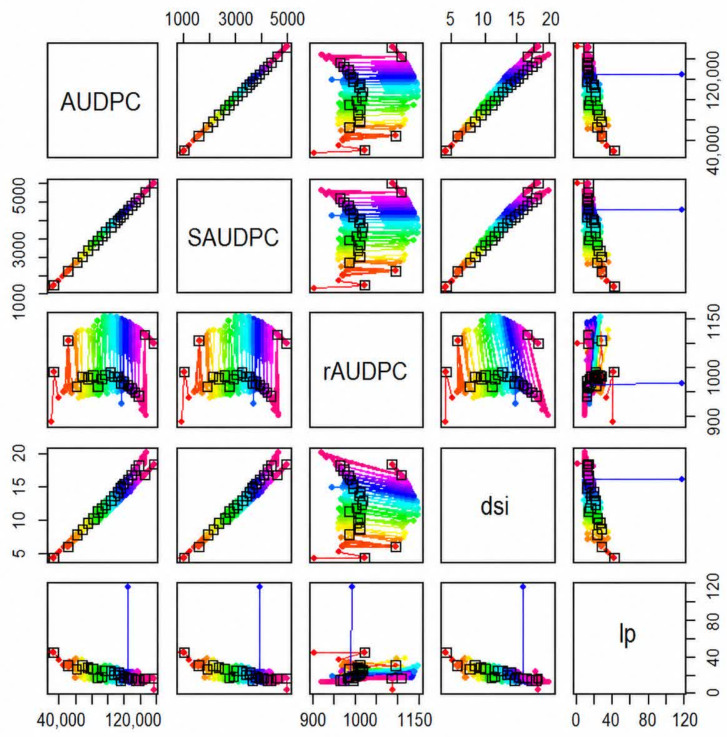
Correlation between disease indices considered in this study. Pair-wise scatter plot with Pearson correlation coefficient (r) between disease AUDPC, SAUDPC, rAUDPC, DSI and LP indices based on data collected on plant phenotypes at 35 days post inoculation with *Fusarium oxysporum* race 1.2 (FOM *1.2*). Pairwise scatter plot matrix (lower and upper boxes) and indices (diagonal boxes) are shown. Different colors indicate different genotypes.

The correlation matrix showed strong and significant positive correlations among AUDPC, SAUDPC, and DSI. rAUDPC also showed a moderate-to-strong positive correlation with these variables. In contrast, LP exhibited a significant negative correlation with AUDPC, SAUDPC, and DSI, while showing a weak correlation with rAUDPC (Figure 4).

### 2.5. Population Structure

**Figure 5:**
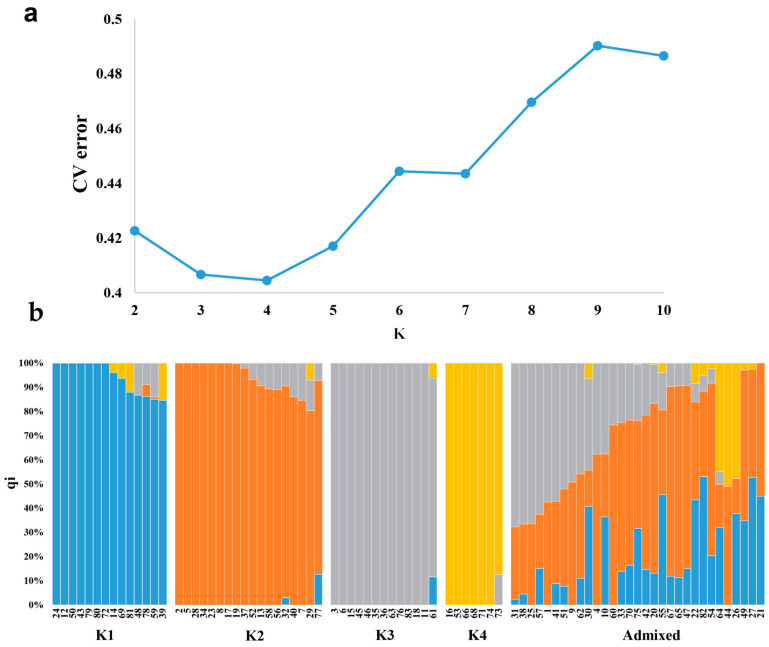
Population structure of studied melon plants based on ADMIXTURE analysis. (**a**) ADMIXTURE cross-validation (CV) error estimates for hypothetical clusters (K) ranging from 1 to 10. (**b**) Population structure of individual melon plants for K = 4. Each vertical bar refers to an individual plant. The length and the color of segments in each bar represent the proportion of the genome (qi) associated with each of the four ancestral populations K1–K4. Individual plants assigned to one of the populations have a membership coefficient (qi) > 0.8 for that population. The remaining individual plants were assigned to the admixed group.

Based on the parametric clustering method, a model with four ancestral populations, K1–K4, was established for the classification of the studied germplasm collection (Figure 5a). According to the results, 52 individual melon plants were assigned to one of the four ancestral populations, while the remaining 31 were allocated to the admixed group, the majority of which were landraces originating from different regions of Iran (Figure 5b). K1 consisted of 14 individual plants, primarily from Iran, including a number of improved cultivars developed in the U.S. and Europe, as well as two individual plants of the Isabelle and Charentais-T genotypes, previously identified as resistant and susceptible based on phenotypic indices. K2 contained 18 individual plants, mostly landraces from different regions of Iran. K3 was composed of 13 individual plants belonging to the *inodorus* type. Seven individual plants fell into K4, mostly originating of Korea and Japan (Figure 5b).

### 2.6. Chromosomal Location of the Identified SNPs and SVs

**Figure 6:**
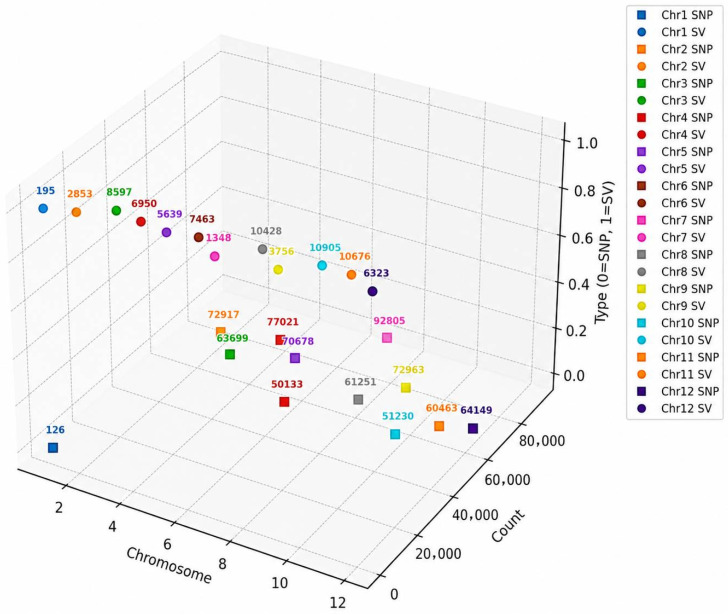
Chromosomal distributions of the identified SNPs and SVs for each chromosome in GBS of melon genotypes.

In this study, on average 3.5 million reads per sample were generated from the sequencing of the GBS libraries. The GBS sequencing generated a high and uniform coverage across the samples. The averaged sequencing depth (mean coverage) ranged from approximately 3.3 to 8.8×, mostly with the average above 5× (Figure S1 and Table S4). In total, 1,938,520 and 340,858 unfiltered SNPs and SVs were detected by the variant calling procedures, respectively. After the final filtering, 737,435 SNPs and 75,133 SVs of high quality were retained and used to perform GWAS analysis. Interestingly, a high level of variation was observed in the distributions of SNPs and SVs across the melon chromosomes. The highest numbers were detected on chromosomes 7 and 10 with 92,805 and 10,905 SNPs and SVs, respectively. For SNPs, the next highest numbers were observed on chromosomes 4 (77,021) and 9 (72,963), whereas for SVs, chromosomes 11 (10,676) and 8 (10,428) ranked next. In contrast, the lowest numbers of SNPs and SVs were detected on chromosome 1, with 126 SNPs and 195 SVs. The number of SNPs and SVs identified for each chromosome are provided in Figure 6. In total, 22,922,715 transitions and 10,016,336 transversions mutations were identified in the melon genome, resulting in a transition/transversion ratio of 2.28.

### 2.7. Average LD Decay

**Figure 7:**
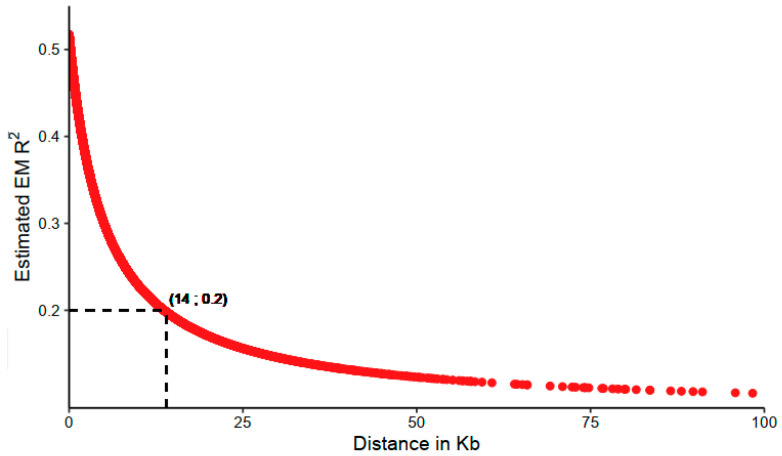
Genome-wide average LD decay plot based on SNP data. On average, R^2^ of 0.2 was assumed to be the lowest threshold to detect LD between two loci, which is 14 kb.

To accurately delineate the LD pattern, SNP markers were used across the genome. Correlation of the allelic state at pairs of different loci, expressed through the pairwise squared correlation coefficient R2, was assumed equal to 0.2 on average. This value was assumed to be the lower threshold to detect LD between two loci, which was reached on average after 14 kb (Figure 7).

### 2.8. Identification of Gene-Associated SNPs on Melon Genome

**Figure 8:**
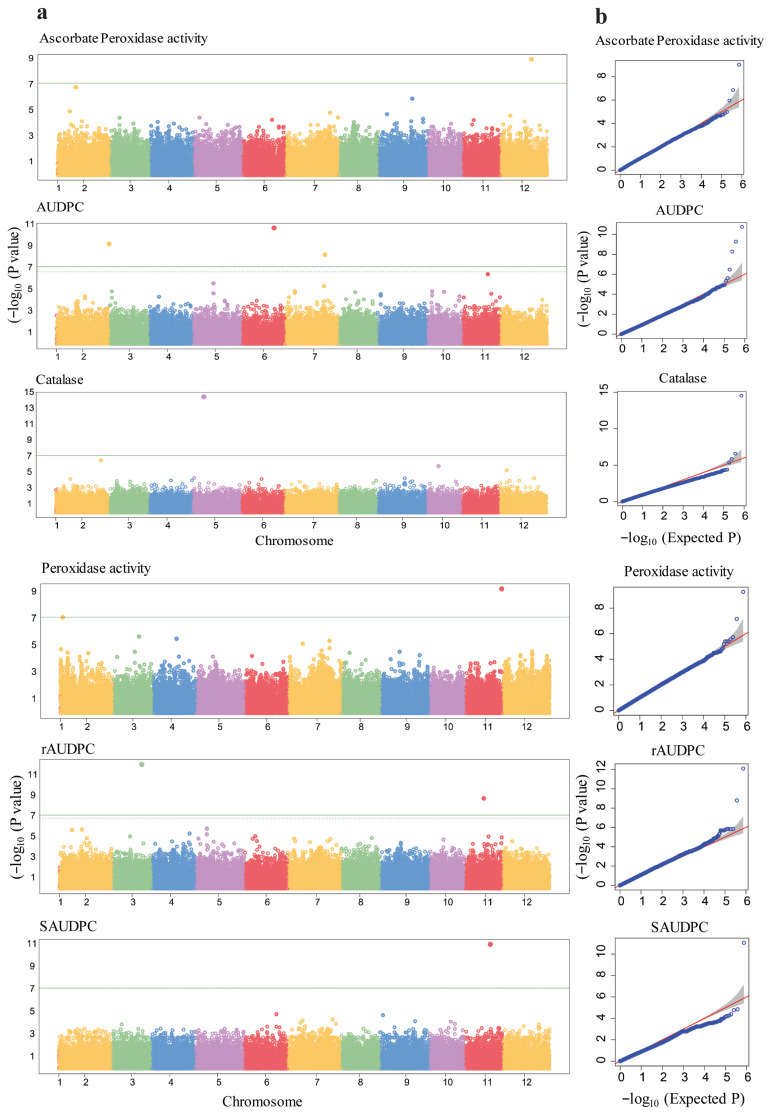
SNP-based Manhattan and Q-Q plots obtained by performing GWAS for ascorbate peroxidase activity, AUDPC, catalase activity, peroxidase activity, rAUDPC, and SAUDPC with the BLINK model in melon plants inoculated with FOM 1.2 (*Fusarium oxysporum* race 1.2). (**a**) Manhattan plots. The green horizontal line indicates a significant FDR-corrected (−log_10_ *p*-value ≥ 7). Dots above green horizontal lines indicate association for FDR-corrected *p*-value > 0.01. (**b**) Q–Q plots relative to the regression models. The red line represents the expected values under a normal distribution.

In total, nine significant SNPs were found by applying the BLINK model for six studied traits (Figure 8 and Table S5). The highest level of association (−log_10_ (*p*-value) = 14.53; FDR *p*-value 2.13 × 10^−9^) was found between catalase activity and the marker S1_7910597, positioned on chromosome 5 and located in the proximity of the MELO3C018228.2.1 and MELO3C018229.2 genes. Nevertheless, no significant homology of known function was detected for these two genes (Tables S5 and S6). On the other hand, the SNP marker S1_21196037, located on chromosome 12, was associated with ascorbate peroxidase activity (−log_10_ (*p*-value) = 9.02; FDR *p*-value = 0.0006) (Tables S5 and S6). Three other SNP loci, significantly associated with AUDPC, were found on chromosomes 2, 6, and 7 (Figure 8 and Tables S5 and S6). Specifically, the marker S1_35407515 on chromosome 2 (−log_10_ (*p*-value) = 9.27; FDR *p*-value = 0.00019) was located within an exon of the mRNA region encoding a multidrug resistance-associated protein 6, and was in the proximity of three genes MELO3C024400.2, MELO3C024399.2.1, and MELO3C024397.2. Whereas the marker S1_21796996 on chromosome 6 (−log_10_ (*p*-value) = 10.75; FDR *p*-value = 1.30 × 10^−5^) was close to the MELO3C028252.2 gene and showed no significant homology with genes of known function (Tables S5 and S6). Marker S1_27041058 was located on chromosome 7 (−log_10_ (*p*-value) = 8.27; FDR *p*-value = 0.0012) within the coding region DUF1682 (a hypothetical protein) in the mRNA region and in proximity to the gene MELO3C016508.2.1 with no significant homology with known function genes (Tables S5 and S6).

The SNP marker S1_24673367 was detected on chromosome 11 and associated with peroxidase activity (−log_10_ (*p*-value) = 9.27; FDR *P*-value = 0.0003). S1_24673367 was also located in the proximity of MELO3C026481.2 and MELO3C026482.2 genes, with unknown function and phosphomannomutase, respectively. (Tables S5 and S6). Two SNP markers, S1_12588460 and S1_19717061, detected on chromosomes 11 and 3, were significantly associated with rAUDPC and linked to the genes encoding LOB domain-containing protein 15 and LEUNIG-like protein, respectively (Table S6). The SNP marker S1_17118860 was detected on chromosome 11 and showed a significant association with SAUDPC (−log_10_ (*p*-value) = 11.04; FDR *p*-value = 6.64 × 10^−6^). No significant homology was detected between S1_17118860 and genes with known functions (Tables S5 and S6).

### 2.9. Identification of Gene-Associated SVs Through GWAS

**Figure 9:**
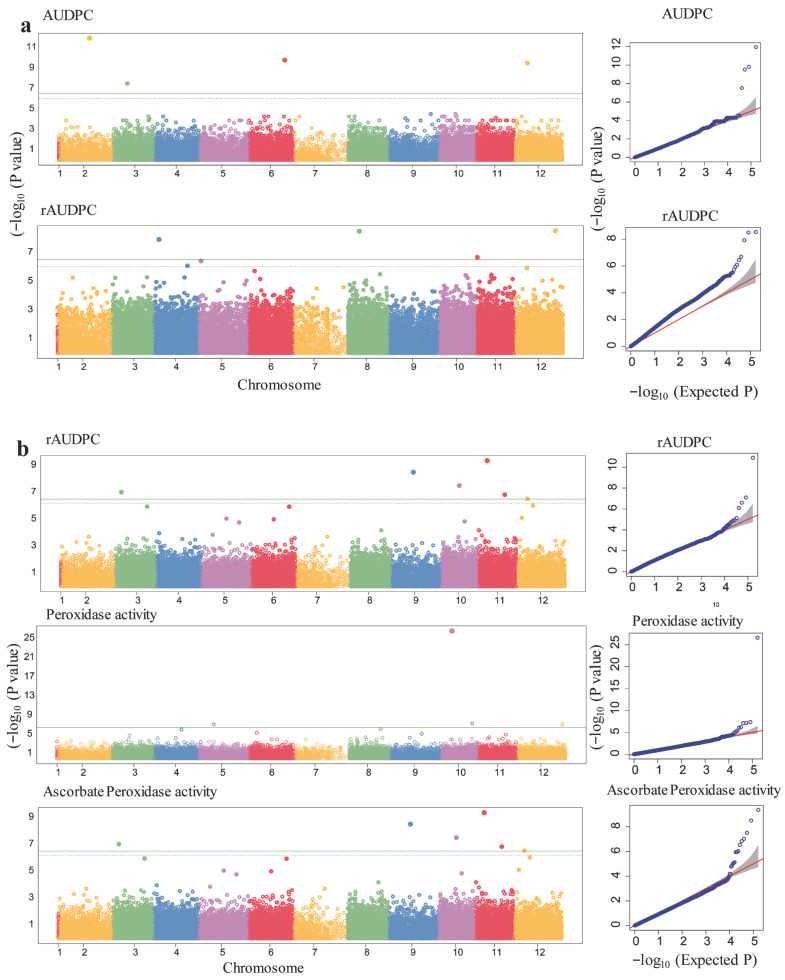
SV-based GWAS Manhattan and Q-Q plots obtained by performing GWAS for AUDPC, rAUDPC, peroxidase activity and ascorbate peroxidase activity using: (**a**) BLINK and (**b**) FarmCPU models in melon plants inoculated with FOM 1.2 (*Fusarium oxysporum* race 1.2). The green horizontal line indicates significant FDR-corrected (−log_10_ *p* ≥ 6.5). Dots above the green horizontal line indicate association for FDR-corrected *p*-value < 0.01. Q–Q plots relative to the regression models. The red line represents the expected values under a normal distribution.

**Figure 10:**
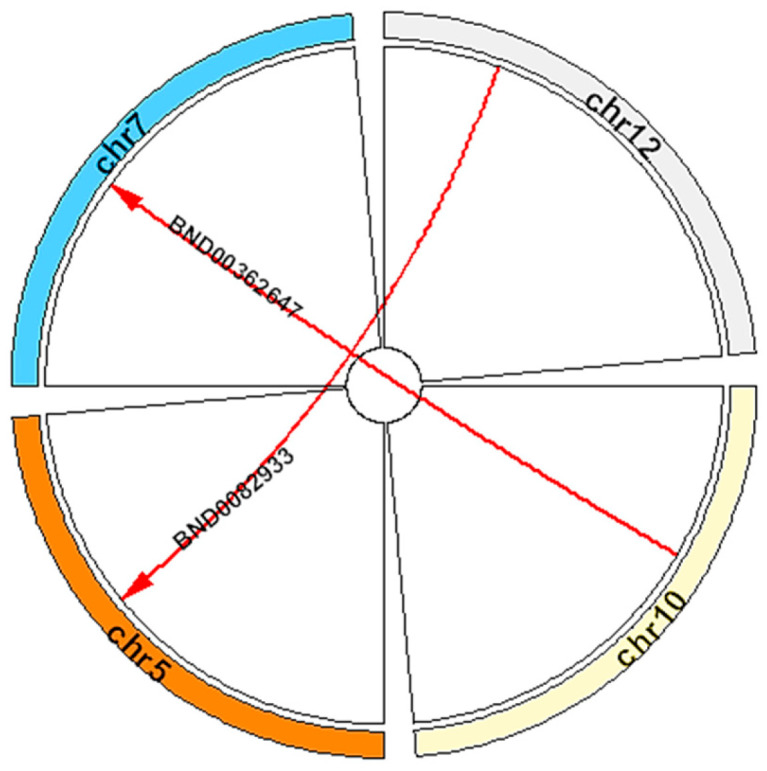
Structural variant (SV) marker translocations from the reference to the studied genomes of melon plants inoculated with FOM 1.2 (*Fusarium oxysporum* race 1.2). Different colors were used to identify chromosomes involved in the detected translocation events. Red arrows denote translocation events between chromosomal regions, and arrowheads indicate the direction of the translocation from the donor chromosome to the recipient chromosome.

Eight significant SVs were found associated with AUDPC and rAUDPC by applying the BLINK model (Figure 9a and Table S7). Additionally, three, four, and six SVs significantly associated with rAUDPC, peroxidase, and ascorbate peroxidase activity, respectively, were identified when applying the FarmCPU model (Figure 9b and Table S8). The SV marker BND0082933 (breakend) from chromosome 12 of the reference genome was translocated to chromosome 5 in our study genome and was associated with rAUDPC (−log_10_ (*p*-value) = 8.53; FDR *p*-value = 0.000257). This SV was detected by both the BLINK and FarmCPU models and was related to a gene coding for the NADH-ubiquinone oxidoreductase B8 subunit. Also, the SV marker BND00362647, located on chromosome 10 of the reference genome, was translocated to chromosome 7 in our study genome and was significantly associated with peroxidase activity (−log_10_ (*p*-value) = 26.59; FDR *p*-value = 4.13 × 10^−22^). This variant was identified using the FarmCPU model and was located close to a gene encoding a polynucleotide adenylyl transferase protein family member (Figure 10). Both BLINK and FarmCPU models consistently detected two shared structural variants (BND00082933 and BND000210837) associated with rAUDPC, suggesting robust and model-independent association signals in these cases. The identified SV-associated genomic regions were considered putative candidate loci in this preliminary analysis.

## 3. Discussion

Melon (*Cucumis melo* L.) remains one of the most widely cultivated cucurbit crops worldwide, yet its production is persistently threatened by soil-borne diseases, foremost among them being Fusarium wilt, caused by *Fusarium oxysporum* f. sp. *melonis* (FOM), which can drastically reduce yield and fruit quality. Melons have been classified into two subspecies, *melo* and *agrestis* [13,38] with 10 and 5 botanical families, respectively [39]. Most commercial melon cultivars are classified under the subspecies *melo*, which includes the botanical varieties cantalupensis, inodorus, and reticulatus. According to previous studies based on SNP markers, melon exhibits limited and often partial resistance to the most destructive FOM race 1.2. [40]. Current findings underscore the complexity of melon breeding for resistance to FOM 1.2, as it is governed by multiple recessive genes and significantly influenced by environmental factors [16]. Based on the relatively moderate heritabilities, several factors are likely involved in resistance and epistatic effects. Hence, the improvement of resistance to FOM 1.2 may be difficult and complicated to achieve through a standard selection procedure, which usually exploits the additive gene effects [41]. In this study, several physiological traits and disease indices were used to evaluate the response of different melon genotypes to FOM 1.2. High LP index and low AUDPC, SAUDPC, rAUDPC, and DSI indicate a high level of disease resistance [41]. Biplot analysis is a powerful method for comparing genotypes based on different criteria [42]. Most of the cultivars proved to be susceptible to FOM 1.2 [15]. The cultivar Isabelle [43] included in the germplasm panel displayed a high level of resistance to FOM 1.2. Moreover, the high levels of antioxidant enzyme activity and phenolic content play important roles in resistance to FOM 1.2 [11]. As shown in Table S3, we found high levels of peroxidase, catalase, and ascorbate peroxidase activities in the studied individual plants, including the Isabelle, Senari1, and Senari2 genotypes. Because antioxidant enzymes contribute to the detoxification of reactive oxygen species produced during pathogen attack, their higher activity may be associated with an efficient early defense response. This is particularly relevant for the Isabelle genotype, which is already known as a resistant genotype, and the elevated enzyme activities observed in this study provide supportive evidence for its resistance. Despite the fact that such biochemical responses are related to general stresses, they may help in the phenotypic evaluation of the resistance to FOM 1.2 among the examined genotypes.

Regardless of deliberations about whether melons originated in Africa or Asia [44], they are highly diverse in morphology and biochemistry [45]. In our genetic analysis based on GBS data, the selected genotypes were assigned to four populations and an admixed group, which might be due to hybridization, though such an interpretation of ADMIXED results should be taken with caution [46] (Figure 5b). Interestingly, both resistant and susceptible genotypes were found within group K1, indicating that the population structure does not perfectly correspond to phenotypic responses and that resistance is likely influenced by multiple loci [16]. Screened individual plants assigned to the K4 population mostly originated of Korea and Japan and included the Senari and Makuwa genotypes, characterized by a high level of resistance to FOM 1.2 [41]. In accordance with previous investigations based on population structure, it was also shown that there is a genetic distinction between Korean and Japanese melon cultivars, including Senari, Makuwa, and other cultivars, in terms of disease resistance to Fusarium wilt disease race 1.2 (Figure 5b).

This population structure has direct implications for the reliability of GWAS results. Because our study involved a relatively small sample size, we applied a stringent minor allele frequency filter (MAF > 10%) to reduce possible statistical noise. Such a threshold is recommended in small-scale GWAS, as it increases statistical power and decreases the likelihood of false-positive associations [47]. Integrating these considerations strengthens the robustness of our detected signals related to FOM 1.2 resistance. The genetic dissection of complex polygenic traits, such as resistance to FOM 1.2 [16], largely benefits from molecular approaches such as GWAS. To effectively scan the genome for marker–trait associations, GWAS is preferably performed based on average marker distances that are largely below the average LD decay distance [30]. In our case, considering the melon genome size of approximately 358 Mb [48], we based our GWAS on marker distances of approximately 1 Kb for SNPs and 3.8 Kb for SVs, which are well below the average LD decay distance of approximately 14 kb.

The description of genes involved in disease resistance mechanisms is vital for a comprehensive understanding of plant defense responses. Concerning the AUDPC index, the association was found with an SNP marker (S1_35407515) in proximity to genes encoding pyridoxal phosphate phosphatase-related, RNA-binding family member, and multidrug resistance-associated protein 6 genes. Also, for AUDPC, a significant association was found with the SNP marker (S1_27041058), which is linked to a hypothetical protein (DUF1682) gene. The pyridoxal phosphate phosphatase-related gene in *Arabidopsis* has been reported to be associated with increased levels of salicylic acid and jasmonic acid, resulting in successful defense responses against pathogens [49]. A key mechanism of activating or deactivating the proteins involved in these networks is phosphorylation catalyzed by protein kinases [50], which are well represented in the identified candidate gene inventory. In *Arabidopsis thaliana*, the upregulation of the *PDX3* gene, which encodes pyridoxine/pyridoxamine 5′-phosphate oxidase, increases the production of pyridoxal 5′-phosphate (PLP), a key cofactor that enhances oxidative stress tolerance. This highlights the critical role of PLP metabolism in plant defense mechanisms under stress conditions [51].

RNA-binding proteins (RBPs) play a pivotal role in plant responses to abiotic stresses by modulating mRNA splicing, stability, and translation. For instance, GRP7 in Arabidopsis enhances cold tolerance through stress-induced splicing regulation [52], while DRG9 in rice improves drought tolerance via mRNA stabilization and stress granule formation [53]. Although no study has yet functionally characterized the role of MRP6 (ABCC6) in Arabidopsis or other plants, accumulating evidence demonstrates that ABC multidrug resistance-associated proteins (ABC-MRPs) play essential roles in plant defense by transporting glutathione conjugates, secondary metabolites, and xenobiotics into the vacuole, thereby enhancing tolerance to both biotic and abiotic stresses [54]. Notably, multidrug resistance-associated protein 6 has been suggested to be rapidly activated upon pathogen perception and may contribute to plant immune responses [55]. Overexpression of the multidrug resistance-associated protein 6 gene has been proposed to enhance plant defense responses against pathogens, and environmental stress could be enhanced by MRP6’s ability to transport harmful substances away from sensitive cellular compartments [56]. Studies have shown that overexpression of related ABC-MRPs, such as AtABCC1 and AtABCC2, enhances tolerance to abiotic stresses like cadmium exposure and salinity in Arabidopsis [57]. Although the biological function of DUF1682 is still unknown, evidence from related DUF families supports its potential role in stress adaptation; for instance, AtRDUF1/2 (DUF1117) in Arabidopsis were shown to be transcriptionally induced under drought and salinity, enhancing ABA-dependent tolerance [58]. Similarly, OsSIDP366 (DUF1644) in rice was validated through expression profiling and transgenic assays to improve drought and salt resistance [59]. However, functional assignment of DUF1682 remains speculative and requires experimental validation.

The association detected between peroxidase activity and the SNP marker S1_24673367 suggests that variation in oxidative stress responses may be partly driven by genetic factors influencing metabolic pathways related to antioxidant production (Table S6). Overexpression of the phosphomannomutase (PMM) gene in *Arabidopsis thaliana* has been shown to enhance tolerance to oxidative stress by increasing ascorbic acid (AsA) biosynthesis, highlighting the pivotal role of PMM in plant stress responses [60]. PMM has also been linked to resistance to *Fusarium oxysporum* f. sp. *cubense* in banana [61]. The LOB domain-containing protein 15 in soybean has been shown to increase resistance to *Phytophthora sojae* [62]. In our study, the SNP marker S1_12588460, associated with the rAUDPC trait, was linked to LOB domain-containing protein 15, suggesting a putative regulatory association in melon. The involvement of LOB domain proteins in defense signaling in other species provides a plausible biological basis, and the known function of this gene in soybean supports the idea that LOB-related pathways may contribute to disease progression and resistance mechanisms in melon. The SV marker BND0082933, associated with the rAUDPC trait, was linked to the NADH-ubiquinone oxidoreductase B8 subunit gene. Overexpression of this gene has been reported to enhance resistance to *Rhizoctonia solani* in tomato, suggesting a potential role in disease defense in melon as well [63]. Publicly available melon transcriptomic datasets were also explored to provide additional support for the candidate genes identified in this study. In the RNA-seq dataset generated by Sebastiani et al., (2017) [43] under *Fusarium oxysporum* f. sp. *melonis* race 1.2 infection conditions (BioProject accession: PRJEB15551), two candidate genes identified in our GWAS showed consistent expression responses. Specifically, MELO3C018228.2.1 was differentially expressed in a resistant cultivar, while MELO3C024400.2 showed a response to Fusarium infection in both resistant and susceptible cultivars. Although, these findings provide additional evidence supporting the potential involvement of these genes in the melon–Fusarium interaction, the identified SNP markers and putative SV-associated genomic regions require further validation in independent populations and through complementary approaches, including transcriptomic analyses across diverse genotypes and functional characterization of candidate genes as putative associations rather than confirmed biological functions in melon. In addition, definitive validation of the predicted structural variants will require higher-coverage whole-genome sequencing and/or long-read sequencing approaches.

## 4. Materials and Methods

### 4.1. Plant Material and Pathogen Inoculation

In the present study, 160 melon genotypes, including landraces and commercial cultivars originating from different areas of Asia, including Iran, Turkey, Afghanistan, Korea, and Japan, were evaluated. Along with these genotypes, Isabelle and Charentais-T genotypes, provided from the French National Research Institute for Agriculture, Food and the Environment (INRAE), were considered as resistant and susceptible control plants, respectively (Table S1). Due to the heterogeneous (non-uniform) genetic nature of the studied melon genotypes, 30 seeds from each of the genotypes were surface-sterilized using a 1% sodium hypochlorite solution for 5 min, followed by two rinses with sterile distilled water. The seeds then sown under controlled glass greenhouse conditions in trays containing 1:2 perlite:cocopeat at 25 ± 2 °C and 60–80% relative humidity [64].

Two weeks after sowing, at the first true leaf stage, 15 individual plants from each genotype (each individual plant were considered independent biological units for evaluations), in a total of 2400 individual melon plants, were artificially inoculated with FOM 1.2 strain Mt13-3a (obtained from Shiraz University, Iran). To prepare the inoculum, the pathogen was cultured in a potato dextrose agar (PDA) medium composed of 300 g potato extract, 20 g dextrose, and 15 g agar for two weeks at 23–25 °C. The conidial suspension was adjusted to a concentration of about 10^6^ spores/mL using a hemocytometer lam. Seedling inoculation was performed as the first true leaf began to emerge, by washing the roots with sterile distilled water and dipping them for 2 min in a fresh FOM 1.2 spore suspension [64]. After inoculation, all the plants were transferred into new trays filled with sterilized 1:2 perlite:cocopeat and maintained in the glass greenhouse at 25 ± 2 °C and 60–80% relative humidity for 35 days [64]. Mock inoculation was performed for the remaining 15 plants from each population by dipping the plants in sterile distilled water.

### 4.2. Phenotyping

Disease evaluation of 2400 melon plants was performed based on disease-related indices, including the area under disease progress curve (AUDPC) [65], the standardized area under disease progress curve (SAUDPC) [66], the relative area under disease progress curve (rAUDPC) [63], the disease severity index (DSI) [66], and the latent period (LP) [67]. These disease-related indices were calculated for each individual plant and used for subsequent multivariate analysis. Subsequently, principal component analysis (PCA) was performed based on these disease-related indices calculated for all individual plants to detect the distribution of disease-response variation. The AUDPC, SAUDPC, and rAUDPC were calculated according to the following equation:

AUDPC = Σi[(xi + xi + 1)/2](ti + 1 − ti),

where xi = mean disease score of each plant at date i, xi + 1 = mean disease score of each plant at date i + 1, and ti + 1 − ti = number of days between scoring date i and scoring date i + 1. SAUDPC = $\frac{AUDPC}{D}$, D = the time of the first note-taking and the time of the last note-taking. rAUDPC = $\frac{SAUDPC}{\overset{-}{Y}}$, $\overset{-}{Y}$ = Average contamination of each treatment. The DSI ranged 1 to 5; 1: no symptoms, 2: wilting of the cotyledons or the first leaf, 3: wilting of two leaves, 4: wilting of three or more leaves and browning of the stem, and 5: dead plant (Perchepied and Pitrat 2004) [16].

### 4.3. Biochemical Evaluation

#### Total Protein Extraction and Enzyme Activity Assay

The individual plants, with a broad range of disease response variation screened through PCA, were used for biochemical assays. Total protein was extracted from the plant roots, and the activities of peroxidase, catalase, and ascorbate peroxidase enzymes were determined. For this purpose, 500 mg root tissue was ground in a pre-chilled mortar with liquid nitrogen and homogenized in an 8 mL solution containing 50 mM potassium phosphate buffer (pH 7.0) and 1% polyvinylpolypyrolidone. The homogenate was centrifuged at 15,000 rpm for 30 min, and the supernatant was collected for enzyme activity assays [68].

Peroxidase activity was assayed in a 300 μL reaction mixture containing 10 mM potassium phosphate buffer (pH 7.0), 8 mM guaicol, and 1.5 μL extracted enzyme. The reaction was initiated by adding 50 μL of 1% H_2_O_2_. The increase in absorbance was recorded within 30 s at 430/470 nm. The unit of peroxidase activity was expressed based on the change in absorbance per min and specific activity as enzyme units per mg soluble protein (extinction coefficient 6.39 mM^−1^ cm^−1^) [68]. Catalase activity was assayed in a 300 μL reaction mixture containing 50 mM potassium phosphate buffer (pH 7.0), 3 μL of enzyme extract, and 1.5 μL of 60 mM H_2_O_2_ to initiate the reaction. The reaction was measured at 240 nm for 3 min, and H_2_O_2_ consumption was calculated using the extinction coefficient, 39.4 mM^−1^ cm^−1^ [68]. Ascorbate peroxidase activity was assayed in a 300 μL reaction mixture containing 50 mM potassium phosphate buffer at pH 7.0, 0.5 mM ascorbate, 5 μL of enzyme extract, and 0.5 mM H_2_O_2_ to initiate the reaction. The decrease in absorbance at 290 nm was measured and monitored for 3 min. The reaction was calculated using the extinction coefficient, 2.8 mM^−1^ cm^−1^ [69].

### 4.4. GBS Library Preparation and Genotyping

DNA from young leaves was extracted using the CTAB method [70], checked for integrity by 1% agarose gel electrophoresis, and quantified by the spectrophotometry method at OD_260_. The restriction enzyme *Ape*KI was used to prepare the GBS library [26]. Paired-end sequencing was performed using the HiSeq2500 platform (Illumina, San Diego, CA, USA).

### 4.5. SNP/SV Calling and Quality Control

The bcftools pipeline (bcftools v1.16 bcftools mpileup -m 2 -F 0.002 -d 1000 -f -b|bcftools call -mv -Ov -O v -o bcftools.vcf) [71] and the Delly software (delly v0.8.7 delly call -g *.bam > delly.vcf) [72] were used for SNP and SV calling/mapping, respectively, using the melon reference genome sequence Melon v4. SNP and SV quality control was performed using TASSEL v. 5 [73]. Specifically, biallelic markers with known chromosomal position were kept and filtered for minor allele frequency (MAF) > 10% and call rate > 0.8 on both datasets. Additionally, INDELs were removed from the SNP dataset previously the applying of filters.

### 4.6. Population Structure Analysis

The structure of populations was evaluated using SNPs in approximate linkage equilibrium, generated by the LD pruning algorithm in PLINK v.1.90p [74] using the filter flag and parameters --indep-pairwise 50 5 0.5. Thus, a genomic window of 50 SNPs was shifted by five SNP at the end of each step, pairwise squared correlation (r^2^) was calculated among SNPs, and the first marker of each pair exceeding the r^2^ threshold of 0.5 was removed. The ADMIXTURE v1.3.0 parametric model [75] was used to analyze ancestral populations (K) in the range 1 to 10. One thousand bootstrap replicates were run to estimate parameter standard errors. The most appropriate number of K was selected based on the lowest cross-validation (CV) error. Genotypes were assigned to a specific ancestral population when their membership coefficient qi was >0.8 for that population, or, alternatively, assigned to the admixed ancestry group.

### 4.7. Estimation of LD Decay

Pairwise linkage disequilibrium (LD) values between SNPs were assessed using PLINK v.1.90p [74]. The formula was applied to describe the LD decay functioning as the mean squared correlation coefficient (R^2^) between pairs of markers with respect to their physical distance [76]. The average LD decay threshold of 0.2 is used as the lowest value to detect two loci considered unlinked [31,77,78].

### 4.8. Genome-Wide Association Studies Analysis

The R package GAPIT v3 [79] was used to carry out GWAS based on either SNP of SV datasets [80]. Bayesian information and linkage-disequilibrium iteratively nested keyway (BLINK) and fixed and random model circulating probability unification (FarmCPU) models were employed alongside VanRaden kinship as the covariance matrix for random genetic effects. The false discovery rate (FDR) correction was used to suggest marker–trait association for *p* < 0.1.

## 5. Conclusions

In summary, this study identified several genomic markers, SNPs or SVs, significantly associated with resistance to FOM 1.2. This information can conveniently be used for melon breeding. In addition, by evaluating a melon germplasm collection for response to FOM 1.2, our study highlighted germplasm displaying a high level of resistance and confirmed the occurrence of a distinct gene pool, originating from the Far East region, harboring genetic sources of resistance to Fusarium wilt disease race FOM 1.2.

## Data Availability

All data generated or analyzed during this study are included in this published article and the Supplementary Information Files. Sequence data that support the findings of this study have been deposited in the NCBI Sequence Read Archive (SRA) under BioProject accession number PRJNA1243015.

## Supplementary Materials

The following supporting information can be downloaded at: https://www.mdpi.com/article/10.3390/plants15142205/s1, Table S1. List of studied melon genotypes from various geographical regions of Asia; Table S2. Disease indices calculated in the screened melon genotypes after inoculation with *Fusarium oxysporum* race 1.2 (FOM 1.2). AUDPC: area under disease progress curve, SAUDPC: standardized area under disease progress curve, rAUDPC: relative area under disease progress curve, DSI: disease severity index, and LP: latent period; Table S3. Activity of peroxidase, catalase, and ascorbate peroxidase enzymes involved in oxidative stress related resistance in the melon plants after inoculation with *Fusarium oxysporum* race 1.2 (FOM 1.2); Table S4. Statistical properties associated with the GBS sequencing data in the screened melon plants using the Illumina HiSeq 2500 paired-end sequencing platform. The table includes total reads, average sequencing depth; Table S5. SNPs associated with six traits (Ascorbate peroxidase activity, AUDPC, Catalase, Peroxidase activity, rAUDPC and SAUDPC) identified using the BLINK model; Table S6. Candidate genes close to SNPs associated with Ascorbate peroxidase activity, AUDPC, Catalase, Peroxidase activity, rAUDPC, and SAUDPC; Table S7. Structural variant (SV) associated with AUDPC and rAUDPC identified using the BLINK model; Table S8. Structural variant (SV) associated with rAUDPC, Peroxidase activity, and Ascorbate peroxidase identified using the FarmCPU model; Figure S1. Mean read depth obtained from genotyping-by-sequencing (GBS) data for screened individual melon plants. Genomic DNA libraries were prepared according to the Illumina library preparation protocol and sequenced on the HiSeq2500 platform using paired-end reads. The average sequencing depth ranged from approximately 3.3× to 8.8× across samples, indicating adequate coverage for downstream analyses.

## Abbreviations

The following abbreviations are used in this manuscript:

GBS	Genotyping By Sequencing
SNPs	Single-Nucleotide Polymorphisms
LD	Linkage disequilibrium
GWAS	Genome-Wide Association Study
BLINK	Bayesian information and linkage disequilibrium iteratively nested keyway
FarmCPU	Fixed and random model Circulating Probability Unification
ROS	Reactive Oxygen Species
NGS	Next-Generation Sequencing
SV	Structural Variant
PDA	Potato Dextrose Agar
AUDPC	Area Under Disease Progress Curve
SAUDPC	Standardized Area Under Disease Progress Curve
rAUDPC	Relative Area Under Disease Progress Curve
DSI	Disease Severity Index
LP	Latent Period
PCA	Principal Component Analysis
CNV	Copy number variation
