# Supplementary material for: Genome-Wide Identification of Melon Single-Nucleotide Polymorphisms and Structural Variations Associated with Resistance to Fusarium oxysporum f. sp. melonis Race 1.2
Source: Plants (Basel). 2026 Jul 19;15(14):2205. doi: 10.3390/plants15142205 (PMC13416282; doi:10.3390/plants15142205)
Supplement: Supplementary file 1 [file plants-15-02205-s001.zip › plants-4329229-supplementary.pdf]

Additional file S1

Genome-wide Identification of Melon Single Nucleotide Polymorphisms and Structural Variations Associated with Resistance to *Fusarium Oxysporum* f. sp. *melonis* Race 1.2

Abolfazl Bozorgmehr [1], Mohammad Sadegh Sabet[1,*], Mohammad Ali Malboobi[2,*], Stefano Pavan[3], Chiara Delvento[3] and Ahmad Moieni[1]

[1]Department of Plant Genetics and Breeding, College of Agriculture, Tarbiat Modares University, Tehran, Iran, P. O. Box 14115-336

[2]Department of Plant Biotech, National Institute of Genetic Engineering and Biotechnology, Tehran, Iran, P. O. Box 14965-161

[3]Department of Soil, Plant and Food Science, University of Bari Aldo Moro, Via Amendola 165/A, Bari 70126, Italy

Correspondence: Mohammad Sadegh Sabet (ms.sabet@modares.ac.ir); Co- Correspondence: Mohammad Ali Malboobi (malboobi@nigeb.ac.ir)

Table S1. List of studied melon genotypes from various geographical regions of Asia

Genotype No.	Genotypes name	Place of collecting	Genotype No.	Genotype name	Place of collecting
1	Aamdchi	Unknown	81	Kheyar Chanbar	Iran (Unknown)
2	Abrame Gorgab	Iran (Esfahan)	82	Kogan Makua	Unknown
3	Afghani	Afghanistan	83	Kori Hybrid F2	Unknown
4	Anjeri	Iran (Unknown)	84	Laki1	Iran (Kuhsorkh)
5	Asgar Aabadi	Iran (Qom)	85	Laki1-2	Iran (Kuhsorkh)
6	Avishni	Iran (Torbate Jam)	86	Laki2	Iran (Kuhsorkh)
7	Birjand	Iran (Birjand)	87	Laki2	Iran (Kuhsorkh)
8	California F1	Unknown	88	Laki Sabz3	Iran (Khorasan)
9	California F2	Unknown	89	Laki Zard	Iran (Khorasan)
10	Choruk siah	Iran (Khorasan)	90	Laki Zard1	Iran (Taybad)
11	INR-C1	INRAE	91	Laki Zard1	Iran (Taybad)
12	Danab	Iran (Khorasan)	92	Laki Zard4	Iran (Taybad)
13	Dastanbo	Iran (Unknown)	93	Majdi	Iran (Yazd)
14	Dastanboi Dezful	Iran (Khuzestan)	94	Mashadi Gerd	Iran (Khorasan)
15	Gabari	Iran (Khorasan)	95	Mashadi Kochak	Iran (Khorasan)
16	Galia Willimorin	Unknown	96	Mashhadi	Iran (Khorasan)
17	Gark	Iran (Kurdistan)	97	Melon Beyzi Shirin	Unknown
18	Garmak Bomi Jiroft	Iran (Jiroft)	98	Niagara × Dablon	Unknown
19	Garmak Esfahan	Iran (Esfahan)	99	Niagara × Laki Mashhad	Iran (Unknown)
20	Garmak Eyvanekey	Iran (Ivanki)	100	Passport	Unknown
21	Garmak Gosht Narenj	Iran (Unknown)	101	Poost Hendevanei	Iran (Bushehr)
22	Gensin × Shahabadi	Korea and Japan	102	Poost Zard	Iran (Semnan)
23	Ghandi	Iran (Kerman)	103	Posteh Hendevanhi	Iran (Bushehr)
24	Ghayenat	Iran (Ghayenat)	104	Qaenat	Iran (Khorasan)
25	Ginscn Makuwa	Korea and Japan	105	Raghm Bandi	Iran (Unknown)
26	Gorgab Isoleh	Iran (Esfahan)	106	Rasenjan	Iran (Unknown)
27	Hoferehi Koghak Poset Mohkam	Iran (Unknown)	107	Samsoori	Iran (Saveh)
28	Hofrehei koochak Poost Mohkam	Iran (Unknown)	108	Samsoori × Ghasri Mahshad	Iran (Khorasan)
29	Honey Did Moghan	Iran (Ardabil)	109	Samsoori × Sosoki Sabz	Iran (Ivanki)
30	Hybrid Khaksari Khatdar	Unknown	110	Saveh × Ferozan	Iran (Unknown)
31	Kaleh Goregi	Unknown	111	Saveh Todeh37	Iran (Saveh)
32	Kaleh Gorgi	Iran (Unknown)	112	Saveh Todeh47	Iran (Saveh)
33	Kanri KiK makuma	Korea and Japan	113	Senari × Niagara	Unknown
34	Kashefi	Iran (Torbat Heydariyeh)	114	Senari1	Korea and Japan
35	Kavum Tohma	Unknown	115	Senari2	Korea and Japan
36	Khaebozeh Talebi sarakhs	Iran (Khorasan)	116	Shadegan	Iran (Khuzestan)
37	Kharbozeh Ali Ghareb	Iran (Khorasan)	117	Shah Abadi	Iran (Unknown)
38	Kharbozeh Ali Gongeh	Iran (Esfahan)	118	Shahpasand F2	Iran (Unknown)
39	Kharbozeh Ananasi Shirin	Iran (Kermanshah)	119	Shapasand F2	Unknown
40	Kharbozeh Bakhrefan Sarakhs	Iran (Khorasan)	120	Showakojane Makuwa × Niagara	Korea and Japan
41	Kharbozeh Boste Sabz Martin	Iran (Esfahan)	121	Soghan	Iran (Kerman)
42	Kharbozeh Chorok Zard	Iran (Khorasan)	122	Sozoki Zard	Iran (Ivanki)
43	Kharbozeh Do Rageh Garm	Iran (Esfahan)	123	Sweet × Heart	Unknown
44	Kharbozeh Ganderz op	Unknown	124	Talebi Barg Nei	Iran (Aran va Bidgol)
45	Kharbozeh Gerin	Unknown	125	Talebi Fyrozan Esfahan	Iran (Esfahan)
46	Kharbozeh Ghaseri	Iran (Khorasan)	126	Talebi Habib Abad Esfahan	Iran (Esfahan)
47	Kharbozeh Ghasri	Iran (Khorasan)	127	Talebi Keshideh	Iran (Unknown)
48	Kharbozeh Ghondorez OP	Afghanistan	128	Talebi Mahali Gorgan	Iran (Gorgan)
49	Kharbozeh Gorgeh Deym	Iran (Ilam)	129	Talebi Resh Baba	Iran (Unknown)
50	Kharbozeh Hoz Sorkh	Iran (Khorasan)	130	Talebi Resh Baba	Iran (Esfahan)
51	Kharbozeh Ivanki	Iran (Ivanki)	131	Talebi Samsori Royan	Iran (Green Biotech company)
52	Kharbozeh Kasaba	Unknown	132	Talebi Shah Abadi	Iran (Esfahan)
53	Kharbozeh Kermani	Iran (Kerman)	133	Tashkandi Bozorg	Iran (Khorasan)
54	Kharbozeh Khaghani	Iran (Khorasan)	134	Taybad1	Iran (Taybad)
55	Kharbozeh Khatoni	Iran (Khorasan)	135	Taybad2	Iran (Taybad)
56	Kharbozeh Latifeh Gorgab	Iran (Esfahan)	136	Thaibad1	Iran (Khorasan)
57	Kharbozeh Mahvolati	Iran (Khorasan)	137	Thaibad2	Iran (Khorasan)
58	Kharbozeh Minoo	Iran (Khorasan)	138	Til Keshedeh	Iran (Khorasan)
59	Kharbozeh Minoo Zero95	Iran (Khorasan)	139	Til Magasi	Iran (Neyshabur)
60	Kharbozeh Mirpanchi Nahavand	Iran (Nahavand)	140	Til Mashadi	Iran (Khorasan)
61	Kharbozeh Rashidi To Ghermez	Iran (Khorasan)	141	Til Zard	Iran (Khorasan)
62	Kharbozeh Robati	Iran (Ivanki)	142	TN-35-7040	Iran Gene Bank
63	Kharbozeh Royan95	Iran (Green Biotech company)	143	TN-62-317	Iran Gene Bank
64	Kharbozeh Sabz Khomeini Shahr	Iran (Esfahan)	144	TN-92-300	Iran Gene Bank
65	Kharbozeh Sefid	Iran (Khaf)	145	TN-92-300	Iran Gene Bank

66	Kharbozeh Shemam	Iran (Tabriz)	146	TN-92-533	Iran Gene Bank
67	Kharbozeh Soski Sabez	Khorasan	147	Us-140	Unknown
68	Kharbozeh Talebi Laki	Iran (Unknown)	148	us14350	Unknown
69	Kharbozeh Til	Iran (Khorasan)	149	Yazdi1	Iran (Yazd)
70	Kharbozeh Torki	Turkiye	150	Yazdi2	Iran (Yazd)
71	Kharbozeh Vahshi Mazandaran	Iran (Mazandaran)	151	Yazdi Ghagh Dar	Iran (Yazd)
72	Kharbozeh Yazdi Bozorg	Iran (Yazd)	152	Zarand	Iran (Kerman)
73	Kharbozeh Yazdi To Ghermez	Iran (Yazd)	153	Zard Ghanari Gerd	Iran (Unknown)
74	Kharbozeh Zard25	Iran (Khorasan)	154	Zard Ghanari Gerd	Iran (Unknown)
75	Kharbozeh Zard Feroazan	Iran (Nahavand)	155	Zard Ghanari Gerd4	Iran (Unknown)
76	Kharbozeh Zard Moatar	Iran (Mashhad)	156	Zard Talai	Iran (Khorasan)
77	Kharbozeh Zard Moshabak Dorosht	Iran (Khorasan)	157	Zemestani	Iran (Khorasan)
78	Kharbozeh Zard Shotori	Iran (Esfahan)	158	Zulf Arose	Iran (Esfahan)
79	Kharbozeh Zard Talai	Iran (Khorasan)	159	**Charentais-T (S)**	**INRAE**
80	Khatooni	Iran (Khorasan)	160	**Isabelle (R)**	**INRAE**

R: Resistant genotype
S: Susceptible genotype

Table S2. Disease indices calculated in the screened melon genotypes after inoculation with *Fusarium oxysporum* race 1.2 (FOM 1.2). AUDPC: area under disease progress curve, SAUDPC: standardized area under disease progress curve, rAUDPC: relative area under disease progress curve, DSI: disease severity index, and LP: latent period

Genotype	AUDPC	SAUDPC	rAUDPC	DSI	LP	Genotype	AUDPC	SAUDPC	rAUDPC	DSI	LP
Afghani	128240	4007.50	1116.84	14.35	13	Laki Zard1	97600	3050.00	997.11	12.23	15
Asgar Aabadi	117920	3685.00	1118.66	13.17	15	Laki Zard4	134480	4202.50	1116.28	15.05	13
Avishni	127360	3980.00	1127.66	14.11	17	Laki1-2	134320	4197.50	1114.96	15.05	13
Birjand	119440	3732.50	1113.20	13.41	13	Mashhadi	144160	4505.00	1109.92	16.23	15
California F2	121600	3800.00	1133.33	13.41	17	Melon Beyzi Shirin	142160	4442.50	1110.62	16.00	13
Danab	114880	3590.00	1130.18	12.70	17	Niagara × Dablon	104800	3275.00	1136.22	11.52	19
Dastanbo	125840	3932.50	1114.20	14.11	13	Niagara × Laki Mashhad	138260	4320.62	1096.27	15.76	9
Gabari	118800	3712.50	986.13	15.05	11	Poost Zard	105380	3293.12	1119.66	11.76	17
Galia Willimorin	138240	4320.00	1112.72	15.52	13	Posteh Hendevanhi	112260	3508.12	1104.41	12.70	13
Garmak Bomi Jiroft	96080	3002.50	1109.62	10.82	13	Raghm Bandi1	88000	2750.00	994.68	11.05	15
Garmak Eyvanekey	112660	3520.62	1108.34	12.70	15	Raghm Bandi2	128480	4015.00	1118.93	14.35	13
Gensin × Shahabadi	112480	3515.00	1004.52	14.00	15	Saveh Todeh37	93600	2925.00	1014.79	11.52	19
Ghayenat	136640	4270.00	1116.76	15.29	13	Saveh Todeh47	112760	3523.75	1089.15	12.94	9
Ginscn Makuwa	116520	3641.25	1085.98	13.41	9	Senari1	39780	1243.12	960.59	5.17	33
Gorgab Isoleh	124240	3882.50	1118.68	13.88	13	Senari2	30660	958.12	904.89	4.23	41
Hoferehi Koghak Poset Mohkam	107060	3345.62	1115.20	12.00	15	Shah Abadi	152560	4767.50	1095.23	17.41	13
INR-C1	136640	4270.00	1116.76	15.29	13	Shapasand F2	128480	4015.00	1118.93	14.35	13
Kaleh Goregi	143680	4490.00	1106.23	16.23	11	Showakojane Makuwa × Niagara	114260	3570.62	1103.64	12.94	13
Kanri KiK makuma	93860	2933.12	1108.06	10.58	13	Sweet × heart	114800	3587.50	1129.39	12.70	15
Kavum Tohma	122320	3822.50	1120.38	13.64	15	Talebi Barg Nei	130240	4070.00	1115.96	14.58	13
Kharbozeh Gerin	140400	4387.50	1113.24	15.76	13	Talebi Fyrozan Esfahan	117660	3676.87	976.66	15.05	9
Kharbozeh Ghaseri	98080	3065.00	983.11	12.47	15	Talebi Fyrozan Esfahan	138800	4337.50	1117.23	15.52	15
Kharbozeh Ghondorez OP	116400	3637.50	981.54	14.82	11	Talebi Habib Abad Esfahan	132080	4127.50	1113.77	14.82	13
Kharbozeh Gorgeh Deym	121920	3810.00	1136.31	13.41	17	Talebi Keshideh	114720	3585.00	999.09	14.35	13
Kharbozeh Latifeh Gorgab	111200	3475.00	1001.27	13.88	13	Talebi Resh Baba	164140	5129.37	1050.59	19.52	9
Kharbozeh Minoo	129040	4032.50	1123.81	14.35	15	Tashkandi Bozorg	120720	3772.50	1125.13	13.41	13
Kharbozeh Minoo Zero 95	101060	3158.12	1118.50	11.29	15	Taybad1	134640	4207.50	1117.61	15.05	13
Kharbozeh Mirpanchi Nahavand	118720	3710.00	1001.11	14.82	17	Til Magasi	103060	3220.62	1117.36	11.52	15
Kharbozeh Sefid	120320	3760.00	1121.40	13.41	13	TN-62-317	92900	2903.12	1121.66	10.35	15
Kharbozeh Shemam	142160	4442.50	1110.62	16.00	13	TN-92-300	109060	3408.12	1114.19	12.23	15
Kharbozeh Soski Sabez	131920	4122.50	1112.42	14.82	13	TN-92-300	88820	2775.62	1123.46	9.88	19
Kharbozeh Talebi Laki	64300	2009.37	1067.48	7.52	13	TN-92-533	138800	4337.50	1117.23	15.52	15

Kharbozeh Torki	110500	3453.12	1107.60	12.47	13	Yazdi Ghagh Dar	97300	3040.62	1123.70	10.82	17
Kharbozeh Vahshi Mazandaran	68040	2126.25	976.92	8.70	15	Zarand	108880	3402.50	1014.78	13.41	19
Kharbozeh Yazdi To Ghermez	112260	3508.12	1104.41	12.70	13	Zard Ghanari Gerd	76480	2390.00	1015.75	9.41	25
Kharbozeh Zard Moatar	124720	3897.50	1123.00	13.88	13	Zard Ghanari Gerd 4	96820	3025.62	1118.16	10.82	15
Kharbozeh Zard Moshabak Dorosht	135200	4225.00	1122.26	15.05	17	Zard Talai	127600	3987.50	1129.79	14.11	17
Kharbozeh Zard25	140560	4392.50	1114.51	15.76	15	Zulf Arose1	136640	4270.00	1116.76	15.29	13
Khatooni	129600	4050.00	1128.68	14.35	19	Zulf Arose2	102000	3187.50	1003.47	12.70	15
Kori Hybrid F2	132560	4142.50	1117.81	14.82	13	**Charentais-T (S)**	91360	2855.00	990.51	11.52	15
Lak1	109840	3432.50	1122.16	12.23	13	**Isabelle (R)**	93380	2918.12	1127.45	10.35	19
Laki Zard	152480	4765.00	1094.66	17.41	13						

R: Resistant genotype
S: Susceptible genotype

Table S3. Activity of peroxidase, catalase, and ascorbate peroxidase enzymes involved in oxidative stress related resistance in the melon plants after inoculation with *Fusarium oxysporum* race 1.2 (FOM 1.2)

Genotype	Peroxidase activity (U/mg)	Catalase activity (U/mg)	Ascorbate peroxidase activity (u/mg)	Genotype	Peroxidase activity (U/mg)	Catalase activity (U/mg)	Ascorbate peroxidase activity (U/mg)
Afghani	5.470	0.018	0.335	Laki Zard1	5.625	0.059	0.107
Asgar Aabadi	1.503	0.017	0.220	Laki Zard4	3.941	0.032	0.523
Avishni	4.672	0.067	0.369	Laki1-2	2.883	0.012	0.117
Birjand	2.076	0.012	0.260	Mashhadi	1.760	0.019	0.284
California F2	3.331	0.019	0.273	Melon Beyzi Shirin	3.375	0.009	0.350
Danab	4.906	0.012	0.326	Niagara × Dablon	1.749	0.014	0.031
Dastanbo	13.884	0.074	0.835	Niagara × Laki Mashhad	3.177	0.010	0.172
Gabari	5.340	0.062	0.101	Poost Zard	0.797	0.020	0.250
Galia Willimorin	3.266	0.006	0.024	Posteh Hendevanhi	5.996	0.013	0.136
Garmak Bomi Jiroft	5.369	0.025	0.112	Raghm Bandi1	2.990	0.026	0.029
Garmak Eyvanekey	5.002	0.023	0.479	Raghm Bandi2	3.037	0.015	0.449
Gensin × Shahabadi	13.233	0.019	0.154	Saveh Todeh37	5.520	0.093	0.127
Ghayenat	3.511	0.017	0.634	Saveh Todeh47	3.746	0.012	0.278
Ginscn Makuwa	7.816	0.012	0.099	Senari1	48.132	0.164	1.392
Gorgab Isoleh	10.822	0.036	0.107	Senari2	27.504	0.113	1.273
Hoferehi Koghak Poset Mohkam	10.512	0.014	0.339	Shah Abadi	11.027	0.077	0.361
INR-C1	11.012	0.055	0.545	Shapasand F2	2.975	0.074	0.092
Kaleh Goregi	6.492	0.016	0.381	Showakojane Makuwa × Niagara	16.493	0.008	0.759
Kanri KiK makuma	35.350	0.098	1.591	Sweet × heart	4.293	0.022	0.277
Kavum Tohma	1.580	0.009	0.084	Talebi Barg Nei	2.231	0.034	0.232
Kharbozeh Gerin	4.514	0.051	0.305	Talebi Fyrozan Esfahan	0.685	0.090	0.232
Kharbozeh Ghaseri	5.375	0.026	0.052	Talebi Fyrozan Esfahan	12.496	0.086	1.857
Kharbozeh Ghondorez OP	6.811	0.052	0.141	Talebi Habib Abad Esfahan	5.541	0.022	0.255
Kharbozeh Gorgeh Deym	5.672	0.026	0.107	Talebi Keshideh	5.113	0.037	0.144
Kharbozeh Latifeh Gorgab	8.949	0.014	0.037	Talebi Resh Baba	8.410	0.003	0.722
Kharbozeh Minoo	4.815	0.012	0.228	Tashkandi Bozorg	7.528	0.053	0.128
Kharbozeh Minoo Zero 95	2.167	0.013	0.123	Taybad1	3.371	0.018	0.233
Kharbozeh Mirpanchi Nahavand	2.154	0.154	0.709	Til Magasi	5.878	0.075	0.586
Kharbozeh Sefid	10.613	0.054	1.857	TN-62-317	26.885	0.080	1.355

Kharbozeh Shemam	3.090	0.001	0.207	TN-92-300	4.217	0.009	0.183
Kharbozeh Soski Sabez	2.280	0.023	0.145	TN-92-300	28.209	0.113	1.273
Kharbozeh Talebi Laki	7.248	0.031	0.088	TN-92-533	26.885	0.107	1.355
Kharbozeh Torki	3.787	0.047	0.199	Yazdi Ghagh Dar	1.817	0.034	0.014
Kharbozeh Vahshi Mazandaran	0.794	0.013	0.192	Zarand	15.558	0.059	0.675
Kharbozeh Yazdi To Ghermez	1.783	0.007	0.118	Zard Ghanari Gerd	3.408	0.023	0.088
Kharbozeh Zard Moatar	6.778	0.030	0.110	Zard Ghanari Gerd 4	13.781	0.086	1.857
Kharbozeh Zard Moshabak Dorosht	11.132	0.058	0.227	Zard Talai	6.378	0.010	0.229
Kharbozeh Zard25	11.027	0.077	0.361	Zulf Arose1	2.742	0.087	0.247
Khatooni	3.626	0.001	0.119	Zulf Arose 2	5.427	0.034	0.471
Kori Hybrid F2	1.615	0.002	0.087	**Charentais-T (S)**	4.310	0.019	0.079
Laki1	2.616	0.013	0.246	**Isabelle (R)**	30.443	0.285	1.857
Laki Zard	3.337	0.134	0.379				

R: Resistant genotype
S: Susceptible genotype

Table S4. Statistical properties associated with the GBS sequencing data in the screened melon plants using the Illumina HiSeq 2500 paired-end sequencing platform. The table includes total reads, average sequencing depth.

Genotype	Total reads	Average depth (X)	Genotype	Total reads	Average depth (X)
Afghani	3,482,924	5.89	Laki Zard1	3,299,470	7.31
Asgar Aabadi	3,379,708	5.59	Laki Zard4	3,228,104	6.11
Avishni	4,086,380	7.09	Laki12	3,600,200	6.79
Birjand	4,040,494	7.80	Mashhadi	2,949,798	6.29
California F2	4,289,582	6.49	Melon Beyzi Shirin	4,333,108	6.03
Danab	2,751,550	6.38	Niagara × Dablon	3,238,297	7.03
Dastanbo	3,659,146	4.84	Niagara × Laki Mashhad	3,967,602	6.48
Gabari	4,443,514	7.05	Poost Zard	3,542,134	5.21
Galia Willimorin	4,322,034	6.3	Posteh Hendevanhi	2,627,444	5.11
Garmak Bomi Jiroft	4,378,793	5.61	Raghm Bandi1	4,064,116	7.08
Garmak Eyvanekey	4,294,154	7.19	Raghm Bandi2	3,954,458	6.31
Gensin × Shahabadi	2,917,323	6.86	Saveh Todeh37	3,820,964	7.01
Ghayenat	3,344,548	5.47	Saveh Todeh47	3,241,428	5.22
Ginscn Makuwa	4,354,030	7.38	Senari1	3,925,443	8.85
Gorgab Isoleh	3,469,471	6.60	Senari2	4,592,330	7.21
Hoferehi Koghak Poset Mohkam	3,185,964	5.94	Shah Abadi	3,562,916	7.14
INR-C1	3,691,882	5.63	Shapasand F2	3,980,428	6.44
Kaleh Goregi	2,533,222	5.48	Showakojane Makuwa × Niagara	2,968,360	5.57
Kanri KiK makuma	3,709,062	6.54	Sweet × heart	1,930,730	4.42
Kavum Tohma	3,086,252	4.47	Talebi Barg Nei	3,503,526	5.11
Kharbozeh Gerin	2,619,326	5.66	Talebi Fyrozan Esfahan	4,580,208	8.09
Kharbozeh Ghaseri	3,783,435	7.14	Talebi Fyrozan Esfahan	2,121,446	5.21
Kharbozeh Ghondorez OP	4,014,520	5.95	Talebi Habib Abad Esfahan	4,273,929	6.97
Kharbozeh Gorgeh Deym	4,083,270	8.37	Talebi Keshideh	3,817,348	7.49
Kharbozeh Latifeh Gorgab	4,701,869	7.04	Talebi Resh Baba	3,795,800	7.01
Kharbozeh Minoo	4,580,804	6.88	Tashkandi Bozorg	3,811,053	7.31
Kharbozeh Minoo Zero 95	4,034,300	6.63	Taybad1	3,986,282	5.08
Kharbozeh Mirpanchi Nahavand	4,102,387	6.59	Til Magasi	3,293,796	5.65
Kharbozeh Sefid	4,046,078	6.31	TN-62-317	3,202,722	5.77
Kharbozeh Shemam	2,604,560	5.60	TN-92-300	2,932,980	6.24

Kharbozeh Soski Sabez	3,212,320	5.64	TN-92-300	4,011,072	3.37
Kharbozeh Talebi Laki	4,416,272	7.54	TN-92-533	4,350,786	7.85
Kharbozeh Torki	2,792,170	6.39	Yazdi Ghagh Dar	2,727,230	5.86
Kharbozeh Vahshi Mazandaran	3,593,139	6.45	Zarand	2,829,642	6.54
Kharbozeh Yazdi To Ghermez	2,075,934	4.15	Zard Ghanari Gerd	3,968,445	5.96
Kharbozeh Zard Moatar	4,004,999	6.41	Zard Ghanari Gerd 4	2,929,378	4.34
Kharbozeh Zard Moshabak Dorosht	4,199,206	7.59	Zard Talai	4,041,474	6.55
Kharbozeh Zard25	3,525,074	6.87	Zulf Arose1	3,476,116	6.95
Khatooni	4,065,935	6.4	Zulf Arose2	3,358,260	5.7
Kori Hybrid F2	2,994,120	5.00	**Charentais-T (S)**	4,334,144	6.30
Laki1	2,904,680	5.01	**Isabelle (R)**	2,363,842	5.21
Laki Zard	3,138,660	6.47			

R: Resistant genotype
S: Susceptible genotype

Table S5. SNPs associated with six traits (Ascorbate peroxidase activity, AUDPC, Catalase, Peroxidase activity, rAUDPC and SAUDPC) identified using the BLINK model.

**Trait**	**SNP**	**Chromosome**	**Position**	***P* value**	**MAF**	**Effect**
Ascorbate peroxidase activity	S1_21196037	12	21196037	9.42 $^{e-10}$	0.48	1.31
AUDPC	S1_35407515	2	35407515	5.27 $^{e-10}$	0.13	- 0.82
	S1_21796996	6	21796996	1.76 $^{e-11}$	0.47	- 1.91
	S1_27041058	7	27041058	5.26 $^{e-9}$	0.19	- 0.47
Catalase	S1_7910597	5	7910597	2.89 $^{e-15}$	0.47	1.65
Peroxidase activity	S1_24673367	11	24673367	5.31 $^{e-10}$	0.48	1.80
rAUDPC	S1_12588460	11	12588460	1.58 $^{e-9}$	0.39	- 0.96
	S1_19717061	3	19717061	7.9 $^{e-13}$	0.43	- 1.06
SAUDPC	S1_17118860	11	17118860	9 $^{e-12}$	0.28	0.88

Table S6. Candidate genes close to SNPs associated with Ascorbate peroxidase activity, AUDPC, Catalase, Peroxidase activity, rAUDPC, and SAUDPC

Trait	SNP	Chromosome	Candidate gene	E value	Candidate gene function
Ascorbate peroxidase activity	S1_ 21196037	12	-	-	-
AUDPC	S1_35407515	2	MELO3C024400.2	-	Pyridoxal phosphate phosphatase-related protein
		2	MELO3C024399.2.1	1^{e-126}	Pyridoxal phosphate phosphatase-related protein
		2	MELO3C024397.2	2^{e-98}	RNA-binding (RRM/RBD/RNP motifs) family protein
		2	MELO3C024398.2.1	0	multidrug resistance-associated protein 6
	S1_21796996	6	MELO3C028252.2	-	Unknown
	S1_27041058	7	MELO3C016507.2.1	0	hypothetical protein (DUF1682)
		7	MELO3C016508.2.1	-	Unknown
Catalase	S1_7910597	5	MELO3C018228.2.1	-	Unknown
		5	MELO3C018229.2	-	Unknown
Peroxidase activity	S1_24673367	11	MELO3C026481.2	-	Unknown
		11	MELO3C026482.2	8^{e-153}	phosphomannomutase
rAUDPC	S1_12588460	11	MELO3C020121.2	1^{e-78}	LOB domain-containing protein 15
	S1_19717061	3	MELO3C029485.2	-	Unknown
		3	MELO3C029692.2	6^{e-7}	LEUNIG-like protein
SAUDPC	S1_17118860	11	MELO3C034435.2	-	Unknown

Note: – indicates that the value was not available or was not determined.

Table S7. Structural variant (SV) associated with AUDPC and rAUDPC identified using the BLINK model

Trait	SV	Chromosome	Position	*P* value	MAF	Effect
AUDPC	BND00067626	12	8872127	3.04^{e-10}	0.43	1.03
	DEL00013481	2	20088042	1.11^{e-12}	0.37	-0.71
	BND00164733	3	9081767	3.06^{e-8}	0.20	0.64
	BND00145165	6	23421535	1.57^{e-10}	0.45	-1.18
rAUDPC	BND00297020	11	930523	2.05^{e-7}	0.26	0.68
	BND00082933	12	26740798	2.91^{e-9}	0.35	0.75
	BND00092092	4	3592899	1.19^{e-8}	0.35	0.79
	BND00210837	8	7492554	3.16^{e-9}	0.40	0.93

Table S8. Structural variant (SV) associated with rAUDPC, Peroxidase activity, and Ascorbate peroxidase identified using the FarmCPU model.

Trait	SV	Chromosome	Position	*P* value	MAF	Effect
rAUDPC	BND00082933	12	26740798	8.04^{e-8}	0.35	0.47
	BND00039297	5	5188928	2.53^{e-7}	0.43	0.54
	BND00210837	8	7492554	1.28^{e-11}	0.40	0.94
Peroxidase activity	BND00362647	10	7027111	2.54^{e-27}	0.44	-1.78
	BND00389475	10	20297569	4.28^{e-8}	0.40	-0.60
	BND00085759	12	29810263	7.83^{e-8}	0.31	0.61
	BND00044256	5	9956978	6.99^{e-8}	0.16	0.45
Ascorbate peroxidase activity	BND00371255	10	11604541	3.07^{e-8}	0.25	0.37
	BND00307002	11	6009714	4.26^{e-10}	0.22	0.52
	BND00328966	11	17585093	1.45^{e-7}	0.46	0.59
	INV00066077	12	7185432	2.92^{e-7}	0.45	-0.60
	INV00157906	3	3854610	9.48^{e-8}	0.26	0.38
	BND00026485	9	14815671	3.07^{e-9}	0.30	0.42

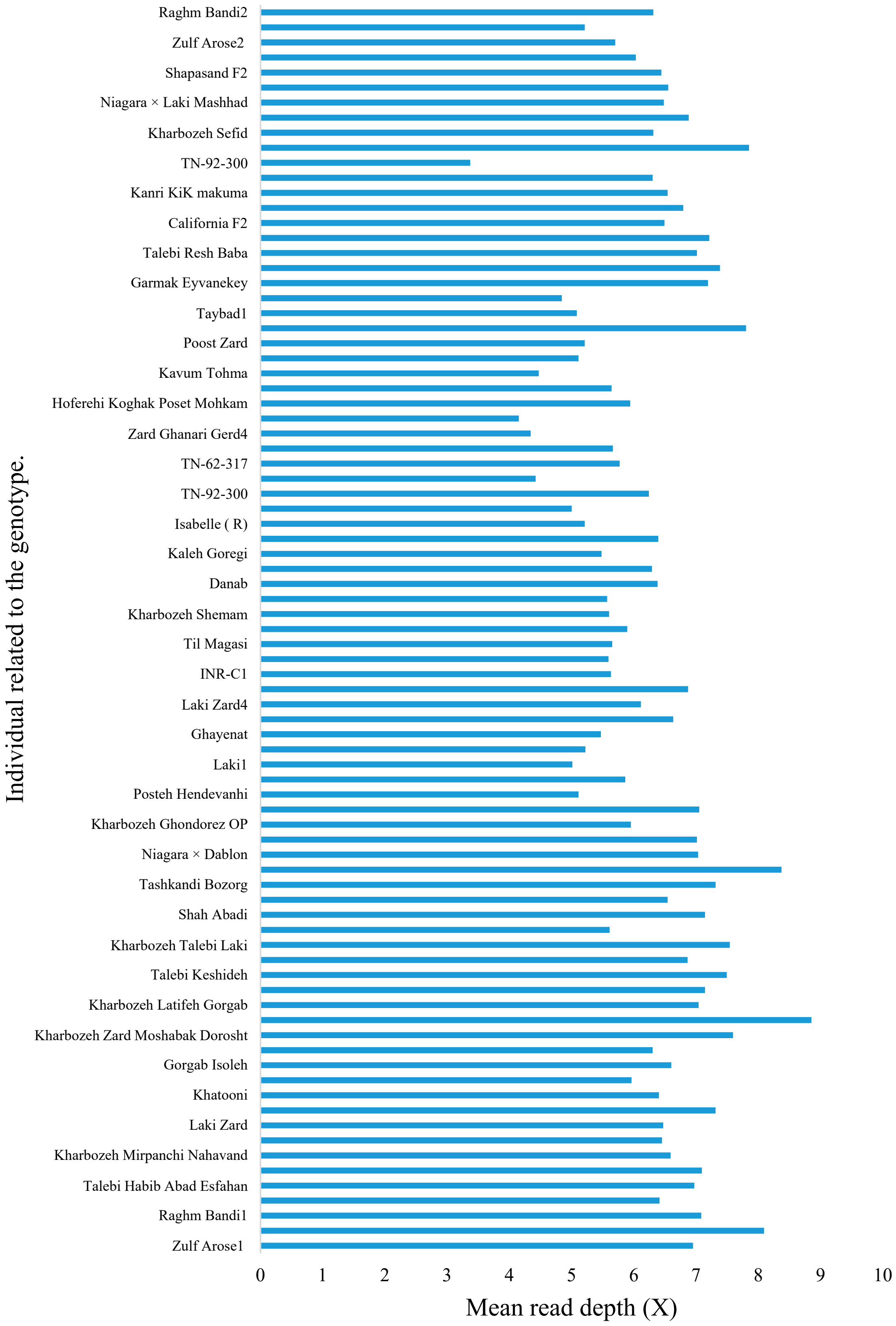
Raghm Bandi2
Zulf Arose2
Shapasand F2
Niagara × Laki Mashhad
Kharbozeh Sefid
TN-92-300
Kanri KiK makuma
California F2
Talebi Resh Baba
Garmak Eyvanekey
Taybad1
Poost Zard
Kavum Tohma
Hoferehi Koghak Poset Mohkam
Zard Ghanari Gerd4
TN-62-317
TN-92-300
Isabelle (R)
Kaleh Goregi
Danab
Kharbozeh Shemam
Til Magasi
INR-C1
Laki Zard4
Ghayenat
Laki1
Posteh Hendevanhi
Kharbozeh Ghondorez OP
Niagara × Dablon
Tashkandi Bozorg
Shah Abadi
Kharbozeh Talebi Laki
Talebi Keshideh
Kharbozeh Latifeh Gorgab
Kharbozeh Zard Moshabak Dorosht
Gorgab Isoleh
Khatooni
Laki Zard
Kharbozeh Mirpanchi Nahavand
Talebi Habib Abad Esfahan
Raghm Bandi1
Zulf Arose1
0
1
2
3
4
5
6
7
8
9
10
Mean read depth (X)
Individual related to the genotype.

Figure S1. Mean read depth obtained from genotyping-by-sequencing (GBS) data for screened individual melon plants. Genomic DNA libraries were prepared according to the Illumina library preparation protocol and sequenced on the HiSeq2500 platform using paired-end reads. The average sequencing depth ranged from approximately 3.3× to 8.8× across samples, indicating adequate coverage for downstream analyses.
